# Antiparkinsonian Efficacy of Guanosine in Rodent Models of Movement Disorder

**DOI:** 10.3389/fphar.2017.00700

**Published:** 2017-10-04

**Authors:** Caio M. Massari, Marc López-Cano, Fabiana Núñez, Víctor Fernández-Dueñas, Carla I. Tasca, Francisco Ciruela

**Affiliations:** ^1^Programa de Pós-graduação em Bioquímica, Centro de Ciências Biológicas, Universidade Federal de Santa Catarina, Florianópolis, Brazil; ^2^Unitat de Farmacologia, Departament de Patologia i Terapèutica Experimental, Facultat de Medicina, Bellvitge Institute for Biomedical Research, Universitat de Barcelona, Barcelona, Spain; ^3^Institut de Neurociències, Universitat de Barcelona, Barcelona, Spain; ^4^Departamento de Bioquímica, Centro de Ciências Biológicas, Universidade Federal de Santa Catarina, Florianópolis, Brazil

**Keywords:** guanosine, Parkinson’s disease, catalepsy, tremor, hemiparkinsonism, dyskinesia

## Abstract

Guanosine (GUO) is a guanine-based purine nucleoside with important trophic functions and promising neuroprotective properties. Although the neuroprotective effects of GUO have been corroborated in cellular models of Parkinson’s disease (PD), its efficacy as an antiparkinsonian agent has not been fully explored in PD animal models. Accordingly, we evaluated the effectiveness of GUO in reversing motor impairments in several rodent movement disorder models, including catalepsy, tremor, and hemiparkinsonism. Our results showed that orally administered GUO antagonized reserpine-mediated catalepsy, reduced reserpine-induced tremulous jaw movements, and potentiated the number of contralateral rotations induced by L-3,4-dihydroxyphenylalanine in unilaterally 6-hydroxidopamine-lesioned rats. In addition, at 5 and 7.5 mg/kg, GUO inhibited L-DOPA-induced dyskinesia in rats chronically treated with a pro-dopaminergic agent. Overall, we describe the therapeutic potential of GUO, which may be effective not only for reversing parkinsonian motor impairments but also for reducing dyskinesia induced by treatment for PD.

## Introduction

Parkinson’s disease (PD) is a neurodegenerative condition of the central nervous system (CNS) characterized by bradykinesia, tremor, and rigidity (Poewe and Mahlknecht, 2009). The disorder, which is secondary to the loss of dopamine neurons in the substantia nigra, affects approximately 1% of the population over the age of 65 years (Meissner et al., 2011). Since the 1970s, the main therapeutic approach has consisted of administrating L-3,4-dihydroxyphenylalanine (L-DOPA) or other dopamine receptor agonists, aiming to reestablish normal function in the affected dopaminergic signaling circuitry (Poewe, 2009). However, adverse effects appear with the long consumption of dopaminergic drugs (Huot et al., 2013), among which dyskinesia -specifically L-DOPA-induced dyskinesia (LID)- is one of most often reported and most likely to impede normal life. Antiparkinsonian drugs are even classified clinically based on their probability of inducing dyskinesia, and it has been shown that rotating these drugs can diminish the appearance of these adverse motor effects. Nevertheless, novel agents are clearly needed to improve the management of PD (Schapira et al., 2006).

Over recent years, new drugs have been developed that not only improve the clinical response to classical drugs but that also alleviate undesired side effects. Among these, purine-based drugs, specifically adenosine A_2A_ receptor (A_2A_R) antagonists, represent realistic and promising non-dopaminergic treatment options (Schapira et al., 2006). The nucleoside guanosine (GUO) is a guanine-based purine that crosses the blood–brain barrier (Jiang et al., 2008) and induces behavioral effects in rodents. GUO has been demonstrated to exert anticonvulsive (Lara et al., 2001), antinociceptive (Schmidt et al., 2010), anxiolytic-like (Almeida et al., 2017), and antidepressant-like effects (Bettio et al., 2014). In addition, it may have trophic and neuroprotective effects in neural cells (Rathbone et al., 1999; Lanznaster et al., 2016), possibly though adenosine receptors modulation (Dal-Cim et al., 2013). Furthermore, GUO can modulate glutamatergic transmission by stimulating its uptake through transporters and increasing glutamine synthetase activity and glutamate turnover, thereby reducing extracellular glutamate levels and protecting from excitotoxicity (Molz et al., 2011; Dal-Cim et al., 2016). Similarly, GUO-induced neuroprotection in ischemia-like models has been shown to promote the reduction of nitroxidative stress, prevent the alteration of mitochondrial membrane potentials (Thomaz et al., 2016), and control the inflammatory response. These effects occur through inhibition of the transcription factor NF-κB translocation to the nucleus (Dal-Cim et al., 2013), and through the reduction of inflammatory cytokines (Hansel et al., 2015).

The biochemical mechanisms responsible for neurodegeneration in PD are oxidative stress, mitochondrial damage, exacerbated inflammatory response, and glutamatergic excitotoxicity (Dexter and Jenner, 2013). Given the effects of GUO, its use offers a promising therapeutic approach. Interestingly, metabolomic analysis in a PD transgenic mouse model showed decreased GUO levels in the brains of adult transgenic mice with concurrent motor symptoms (Chen et al., 2015). Moreover, reduced striatal GUO levels have been observed after reserpine treatment (Loeffler et al., 1998). The administration of reserpine to rodents gives the classic acute pharmacological model of PD by creating a transient parkinsonian-like state (Duty and Jenner, 2011). Reserpine inhibits vesicular monoamine transport in the CNS, leading to monoamine depletion and motor impairments that resemble PD (e.g., hypokinesia, catalepsy, and oral tremor) (Leão et al., 2015).

In this study, we investigated the pharmacological use of GUO in animal models with motor impairments that resemble PD. Locomotor activity and the effects of GUO were investigated in mice with reserpine-mediated catalepsy and reserpine-induced tremulous jaw movements (TJMs). Contralateral rotations induced by L-DOPA in unilaterally 6-OHDA-lesioned rats were also assessed. Finally, we tested the effect of GUO in hemiparkinsonian rats on the development of LID. We aimed to provide evidence in support of GUO as a novel agent for improving the management of PD.

## Materials and Methods

### Animals

Male Swiss albino mice (30–50 g; from the animal facility of the Federal University of Santa Catarina, Florianópolis, Brazil) and Sprague–Dawley rats (240–250 g; Charles River Laboratories, L’Arbresle, France) were used. Animals were housed in standard cages with free access to food and water, and were maintained under controlled standard conditions (12 h dark/light cycles starting at 7:30 a.m., 22°C temperature, and 66% humidity). All manipulations were carried out between 0900 and 1600 h. Procedures in this study were performed in accordance with relevant guidance from the National Institute of Health Guide for the Care and Use of Laboratory Animals (NIH Publications no. 80-23), the Guide for the Care and Use of Laboratory Animals (Clark et al., 1997), and European Union directives (2010/63/EU). The ethics committees of the relevant institutions (CEUA/UFSC and CEEA/UB) approved the protocol. Efforts were made to minimize suffering and reduce the number of animals used in the experiments.

### Drugs

Reserpine (Sigma-Aldrich, St. Louis, MO, United States) was dissolved in 0.1% acetic acid for subcutaneous (s.c.) administration. GUO (Sigma-Aldrich) was dissolved in saline (NaCl 0.9%) containing 0.5% methylcellulose for oral (p.o.) administration. The 6-hydroxydopamine (6-OHDA; Sigma–Aldrich) was dissolved in a saline solution containing 0.05% ascorbic acid. DL-serine 2-(2,3,4-trihydroxybenzyl) hydrazide hydrochloride (benserazide; Sigma-Aldrich) and 3,4-Dihydroxy-L-phenylalanine (L-DOPA; Abcam Biochemicals, Cambridge, United Kingdom) were dissolved in saline for intraperitoneal (i.p.) administration.

### Assessment of TJMs

**FIGURE 1:**
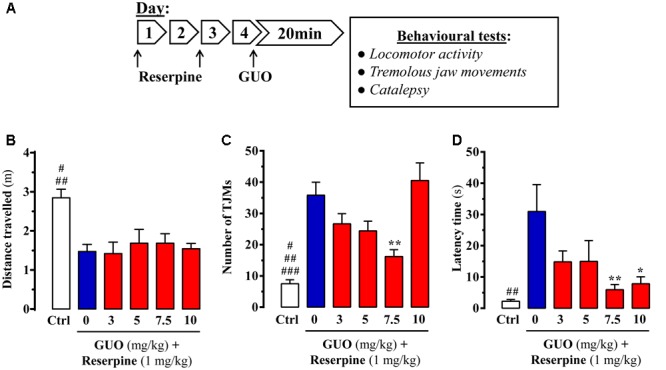
Effect of guanosine (GUO) on reserpine-induced motor disturbances in mice. **(A)** Treatment schedule depicting the administration regimen of reserpine (1 mg/ml; s.c.), guanosine (GUO, 0, 3, 5, 7.5, 10 mg/kg, p.o.) and behavioral testing. **(B)** Spontaneous locomotor activity of mice treated with saline (control mice = Ctrl), or GUO (3, 5, 7.5, or 10 mg/kg, p.o.) after reserpine administration (see **A**) was evaluated in the open-field test. The distance traveled (m) was measured during 10 min. Results are presented as means + SEM (*n* = 9–10 animals). ^#^*P* < 0.05 and ^##^*P*< 0.01 one-way ANOVA with Tukey’s *post hoc* test when compared to 5 and 7.5 mg/kg GUO (^#^), and to 0, 3, and 10 mg/kg GUO (^##^). **(C)** Reserpine-induced orofacial dyskinesia evaluated by tremulous jaw movements (TJMs) frequency during 10 min. Results are presented as means + SEM (*n* = 6 animals). ^#^*P* < 0.05, ^##^*P* < 0.01, and ^###^*P* = 0.001 one-way ANOVA with Tukey’s *post hoc* test when compared to 5 mg/kg GUO (^#^), to 3 mg/kg GUO (^##^) and to 0 and 10 mg/kg GUO (^###^). ^∗∗^*P* < 0.01 one-way ANOVA with Dunnett’s *post hoc* test when compared to vehicle-treated (0 mg/kg GUO) animals. **(D)** Reserpine-induced catalepsy in mice evaluated by the latency scape in the bar test. Results are presented as means + SEM (*n* = 9 animals). ^##^*P* < 0.01 one-way ANOVA with Tukey’s *post hoc* test when compared 0 mg/kg GUO. ^∗^*P* < 0.05 and ^∗∗^*P* < 0.01 one-way ANOVA with Dunnett’s *post hoc* test when compared to 0 mg/kg GUO.

Mice were administered reserpine (1 mg/kg, s.c.) or vehicle (0.1% acetic acid solution) twice at an interval of 48 h. GUO (3, 5, 7.5, or 10 mg/kg; p.o.) was administrated 20 min before behavioral testing and 24 h after the last injection of reserpine (**Figure 1A**). To quantify the occurrence of oral dyskinesia, mice were placed individually in a glass cylinder (13 cm diameter) and hand-operated counters were used to count TJM frequency. Mirrors were placed under the floor and behind the back wall of the cylinder to allow observation when the animal faced away from the observer. TJMs were defined as rapid vertical deflections of the lower jaw that resembled chewing, but were not directed at any particular stimulus (Salamone et al., 1998). If TJM occurred during a period of grooming, they were discounted. The incidence of these oral movements was measured continuously for 10 min.

### Catalepsy Trial

After treatment with reserpine alone or reserpine plus GUO (**Figure 1A**), catalepsy behavior was assessed by placing the forepaws of mice on a horizontal bar (6 mm diameter) positioned at 4.5 cm above the bench surface. The duration of catalepsy, which was defined as an immobile posture, was measured while the animal kept both forepaws on the bar, with a cut-off maximum of 180 s. Three trials were carried out and the results were analyzed using the mean value of the three trials, as adapted from Santos et al. (2013).

### Spontaneous Locomotor Activity

The spontaneous locomotor activity of mice after reserpine or reserpine plus GUO treatment was tested in the open-field test. The apparatus consisted of an acrylic box measuring 45 cm × 45 cm × 45 cm, with each mouse placed in the center and recorded for 10 min with a video camera system. The distance traveled by each animal was analyzed using Bonther Activity Monitoring software (Bonther, Co., Brazil).

The spontaneous locomotor activity of rats was tested in an open-field Plexiglas^®^ arena box measuring 1 m × 1 m × 1 m. Each rat was placed in the center and recorded for 5 min, as described above.

### Hemiparkinsonian Animal Model

Experimental hemiparkinsonism was induced in rats by unilateral injection of 6-OHDA in the medial forebrain bundle, as previously described (Fernández-Dueñas et al., 2015). Rats were stereotaxically injected with 6-OHDA (8 μg of 6-OHDA in 4 μL of saline containing 0.05% ascorbic acid) at anterior–posterior (AP; -2.2 mm), medial–lateral (ML; -1.5 mm), and dorsal–ventral (DV; -7.8 mm) locations with respect to the bregma (Paxinos and Watson, 2007). To minimize damage to noradrenergic neurons, rats were pretreated with desipramine hydrochloride (10 mg/kg, i.p.) 20 min before surgery.

Three weeks later the extent of dopamine deafferentation was checked by assessing the rotating behavioral response to L-DOPA administration. In brief, rats were injected with L-DOPA (50 mg/kg, i.p.) in the presence of benserazide hydrochloride (25 mg/kg, i.p.), an inhibitor of DOPA decarboxylase that minimizes peripheral metabolization of L-DOPA, and the number of full contralateral turns were recorded during a 2 h period. Dopamine deafferentation was considered successful in animals made at least 200 net contralateral rotations.

Thereafter, animals were housed for 3 weeks before being used in the behavioral analyses. GUO was administered orally in a vehicle (0.5% methylcellulose and 2% DMSO) 40 min before benserazide (25 mg/kg; i.p.). Subsequently, L-DOPA (6 mg/kg; i.p.) was delivered after 20 min. The animals were then placed in the rotametry chambers, as previously described (Hodgson et al., 2009), and the number of contralateral rotations was recorded over a 2 h period.

### LIDs and Abnormal Involuntary Movements Rating

L-DOPA-induced dyskinesia were triggered in hemiparkinsonian rats by twice daily administration of L-DOPA (6 mg/kg, i.p.) plus benserazide hydrochloride (15 mg/kg, i.p) for 22 consecutive days. L-DOPA-induced abnormal involuntary movements (AIMs) were scored by a blinded experimenter following a previously described rat dyskinesia scale (Winkler et al., 2002). In brief, rats were injected with L-DOPA, placed in individual transparent plastic cages, and observed every 20 min for 220 min. Three AIM subtypes were monitored (i.e., axial, forelimb, and orolingual) and their respective severity scored from 0 to 4, as previously described (Winkler et al., 2002). Enhanced manifestations of otherwise normal behaviors, such as rearing, sniffing, grooming, and gnawing, were not included. AIM ratings were performed on treatment days 1, 7, 14, and 22 during the chronic L-DOPA administration phase. We calculated integrated AIM scores for each animal and behavioral session using the sum of all three AIM subtypes. AIM was also expressed as an area under the curve (AUC) analysis.

### Data Analysis

Data are represented as means ± SEM. Comparisons among experimental and control groups were performed by one-way analysis of variance (ANOVA) followed by Dunnett’s *post hoc* test when comparing GUO treatments or Tukey’s *post hoc* test when comparing to internal control within the behavioral test, if any. Statistical significance was accepted when *P* < 0.05.

## Results

### GUO Modulation of Reserpine-Induced Motor Disturbances

Reserpine administration to mice was performed to evaluate the ability of GUO to counteract reserpine-mediated changes (**Figure 1A**). We first determined the change in spontaneous locomotor activity. While it was significantly reduced by reserpine administration, acute GUO treatment (3, 5, 7.5, or 10 mg/kg, p.o.) was unable to reverse the change [*F*_(4,43)_ = 0.2241, *P* = 0.9234] (**Figure 1B**).

Next, we assessed the ability of GUO to reduce reserpine-induced TJMs, a parameter known to ameliorate by antiparkinsonian drugs (Collins-Praino et al., 2011). Reserpine-administered animals did, indeed, show a significant increase in TJMs that was partially blocked by GUO administration (**Figure 1C**). One-way ANOVA revealed significant differences between the GUO-treated reserpinized mice [*F*_(4,23)_ = 5.603, *P* = 0.0027], with a significant reduction in reserpine-mediated TJMs observed at 7.5 mg/kg GUO (*P* < 0.01) (**Figure 1C**). Interestingly, treatment with 10 mg/kg of GUO was unable to preclude reserpine-induced TJMs, thus resulting in a U-shaped dose-dependent GUO activity (**Figure 1C**).

Finally, reserpine-induced catalepsy was assessed as an experimental model of akinesia and bradykinesia (Duty and Jenner, 2011). Reserpine treatment induced a cataleptic state, as measured by the latency time of mice to move forepaws from the bar in the bar test, and acute GUO treatment significantly attenuated this increase in latency time [*F*_(4,40)_ = 3.518, *P* = 0.0149] (**Figure 1D**). GUO induced significant reductions in reserpine-mediated catalepsy at doses of 7.5 mg/kg (*P* < 0.01) and 10 mg/kg (*P* < 0.05) (**Figure 1D**).

Overall, although GUO did not reverse reserpine-induced locomotor activity depression, it did ameliorate TJM and catalepsy symptoms in reserpinized mice. Therefore, we considered that antiparkinsonian efficacy was shown in this classical pharmacological animal model of acute PD.

### Antiparkinsonian Effect of GUO in 6-OHDA-Lesioned Rats

**FIGURE 2:**
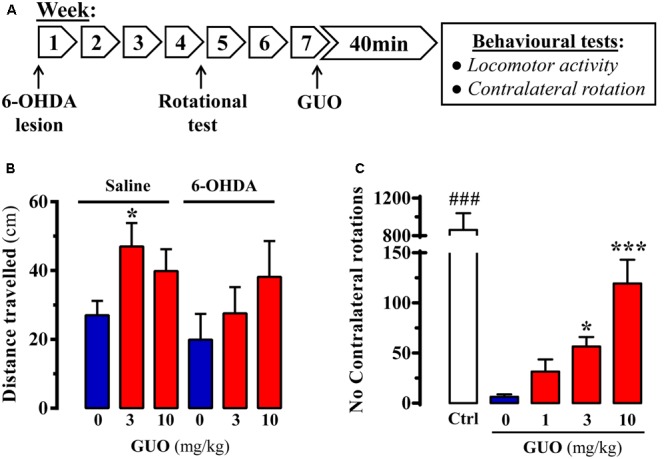
Effect of guanosine (GUO) on hemiparkinsonian rats. **(A)** Treatment schedule depicting the 6-OHDA lesion, rotational test (see section “Materials and Methods”) and the administration regimen of guanosine (GUO, 0, 1, 3, 10 mg/kg, p.o.) and behavioral testing. **(B)** Total distance traveled in the open-field test by either saline- or 6-OHDA-lesioned rats administered with L-DOPA after GUO treatment (3 or 10 mg/kg, p.o.). The distance traveled (cm) was measured during 5 min. Values correspond to the mean ± SEM (*n* = 12). ^∗^*P* < 0.05 one-way ANOVA with Dunnett’s *post hoc* test when compared to 0 mg/kg GUO. **(C)** GUO-mediated potentiation of L-DOPA-induced contralateral rotations in 6-OHDA-lesioned rats. The number of contralateral rotations in 6-OHDA-lesioned rats orally administered with vehicle or GUO (1, 3, or 10 mg/kg) was monitored during a 2 h period. The control group (Ctrl) was administered with L-DOPA (50 mg/kg, i.p.). Values correspond to the mean ± SEM (*n* = 10). ^###^*P* < 0.001 one-way ANOVA with Tukey’s *post hoc* test when compared to 0, 1, 3, and 10 mg/kg GUO. ^∗^*P* < 0.05 and ^∗∗∗^*P* < 0.001 one-way ANOVA with Dunnett’s *post hoc* test when compared to 0 mg/kg GUO.

After assessing the effects of GUO on reserpinized mice, we evaluated its effectiveness in unilateral 6-OHDA-lesioned rats, a classic animal model of experimental parkinsonism based on toxin-mediated destruction of the dopaminergic nigrostriatal pathway (Schwarting and Huston, 1996). Accordingly, we evaluated the impact of acute GUO treatment on spontaneous locomotor activity and contralateral rotation in 6-OHDA-lesioned rats (**Figure 2A**). First, we assessed spontaneous locomotor activity of control and hemiparkinsonian animals. While in 6-OHDA-lesioned animals GUO did not have any effect, in saline-lesioned mice the one-way ANOVA analysis revealed a significant GUO-induced increase in spontaneous locomotor activity at 3 mg/kg (*P* < 0.05) (**Figure 2B**). Overall, GUO was unable to potentiate spontaneous locomotor activity in 6-OHDA-lesioned rats.

In the hemiparkinsonian animal model, asymmetric motor behavior is observed following dopaminergic treatment (i.e., L-DOPA) because of unilateral dopamine depletion in the nigrostriatal pathway (Duty and Jenner, 2011). Interestingly, when using submaximal doses of L-DOPA, it is possible to potentiate contralateral rotations with other pro-dopaminergic drugs (e.g., A_2A_R antagonists) (for review, see Vallano et al., 2011). Therefore, we determined whether GUO could promote contralateral rotations in 6-OHDA-lesioned animals with submaximal (6 mg/kg) L-DOPA dosing in hemiparkinsonian rats (**Figure 2C**). GUO administration alone, up to 10 mg/kg, did not result in asymmetric turning behavior in 6-OHDA-lesioned rats (data not shown). However, GUO did dose-dependently induce contralateral turning behavior when administrated before the subthreshold dose of L-DOPA (**Figure 2C**). One-way ANOVA revealed significant differences between GUO treatments [*F*_(3,36)_ = 11.65, *P* < 0.001] (**Figure 2C**), with GUO inducing significant contralateral rotations at 3 mg/kg (*P* < 0.05) and 10 mg/kg (*P* < 0.001) (**Figure 2C**). Overall, GUO enhanced the effects of L-DOPA with a minimum efficacious oral dose of 3 mg/kg.

### Antidyskinetic Effect of GUO in the LID Rat Model

**FIGURE 3:**
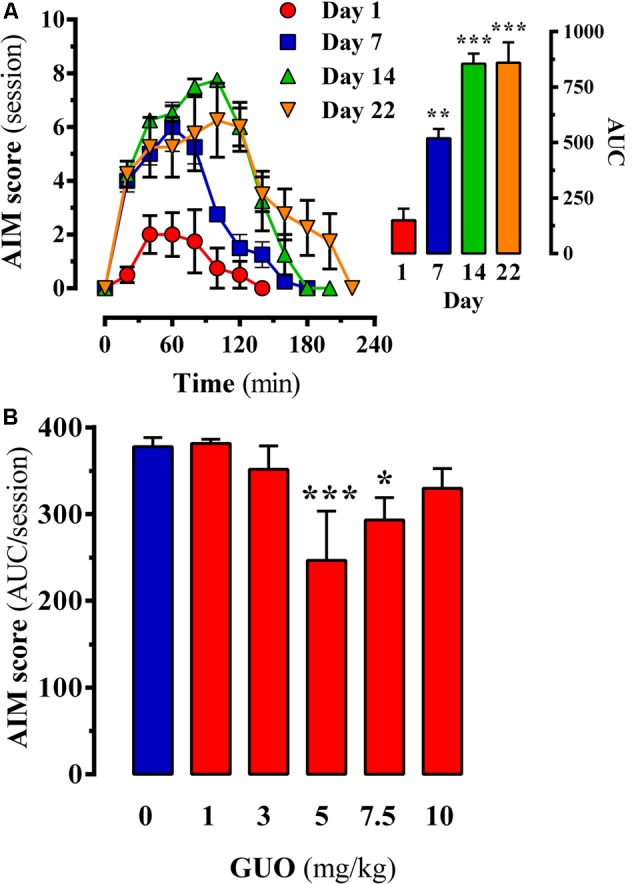
Effect of guanosine (GUO) on dyskinetic rats. **(A)** Development of L-DOPA induced motor side effects (i.e., LIDs) following chronic (22 days) L-DOPA (6 mg/kg) administration. AIMs score was measured during a 220-min session on days 1, 7, 14, and 22 immediately after the corresponding daily L-DOPA injection. **(B)** LIDs attenuation in chronic (22 days) L-DOPA (6 mg/kg) administered rats following GUO administration. The total AIMs score AUC obtained over 220 min following co-administration of L-DOPA (6 mg/kg) plus vehicle or GUO (1, 3, 5, 7.5, or 10 mg/kg) are presented as mean score ± SEM (*n* = 6). ^∗^*P* < 0.05 and ^∗∗∗^*P* < 0.001 one-way ANOVA with Dunnett’s *post hoc* test when compared to 0 mg/kg GUO.

Chronic L-DOPA use in PD is associated with the development of LIDs. Therefore, we assessed the potential antidyskinetic activity of GUO after inducing LIDs in 6-OHDA-lesioned rats through chronic L-DOPA administration and monitoring for the emergence of AIMs over time (**Figure 3A**). AIM severity significantly (*P* < 0.01) increased after 1 week of L-DOPA treatment (**Figure 3A**, inset, day 7), thus LID increased during the first 40 min L-DOPA post-injection and remaining elevated for an additional 40 min (**Figure 3A**, day 7). After 2 weeks of L-DOPA treatment, the increase in LID was higher (*P* < 0.001) (**Figure 3A**, inset, day 14) and sustained in time (i.e., 150 min) (**Figure 3A**, day 14). Finally, after 3 weeks of L-DOPA administration a similar AIMs increase and sustained LID incidences were observed (**Figure 3A**, day 22), thus reaching a LID plateau. Interestingly, the observed time-course in our LID animal model resembled the so-called peak-dose dyskinesia in PD (Fahn, 2000). Thus, we showed that GUO treatment produced a U-shaped dose-dependent antidyskinetic activity in animals administered with L-DOPA for 3 weeks (**Figure 3B**). One-way ANOVA revealed significant differences between GUO treatments [*F*_(5,47)_ = 11.65, *P* = 4.866] (**Figure 3B**), with significantly maximal GUO-induced antidyskinetic activity observed at 5 mg/kg (*P* < 0.001) and 7.5 mg/kg (*P* < 0.05) (**Figure 3B**). Overall, GUO showed antidyskinetic activity in the LID animal model.

## Discussion

Given that dopamine replacement is the first line therapy in PD, treatment with L-DOPA or dopamine agonists (i.e., ropinirole, pramipexole, apomorphine) is the mainstay of clinical management (Poewe and Mahlknecht, 2009). Unfortunately, while dopamine-targeted therapies properly address PD-associated motor disturbances, they also have considerable acute and chronic side effect, including hallucinations, constipation, nausea, somnolence, on/off effects, and dyskinesia (Eggert et al., 2008). In addition, these therapies not only show a progressive decline in efficacy over time but they also do not address common mood, postural instability, or cognitive disturbances. Thus, approaches that indirectly modulate dopaminergic neurotransmission have emerged as potential alternatives to handle side effects associated with PD therapy (Fox et al., 2008).

In this study, we have shown the effectiveness of GUO, a naturally occurring guanine-based purine nucleoside, in three rodent models of impaired movement: (1) the reserpine-induced TJM and catalepsy model in mice, (2) the hemiparkinsonian model of PD in rats, and (3) the LID model in rats. Although GUO was unable to improve spontaneous locomotor activity in reserpine-treated mice, it did ameliorate TJMs and catalepsy in those mice. In addition, GUO potentiated L-DOPA-induced contralateral rotations in unilaterally 6-OHDA-lesioned rats and showed antidyskinetic efficacy in the LID model. Collectively, these results support the hypothesis that GUO has potential use in PD management, including for reducing dyskinesia when used in combination with L-DOPA.

Guanosine has been shown to exert neuroprotective effects in cellular models of PD. For instance, dopaminergic neurons differentiated from human SH-SY5Y neuroblastoma cells were shown to be protected from 6-OHDA-induced toxicity by GUO treatment (Giuliani et al., 2012). The protective effects of GUO were also observed in C6 glioma cells (as a model of astrocytes) when incubated with 6-OHDA (Giuliani et al., 2014). Similarly, GUO neuroprotection has been evidenced against other PD-related toxins, such as 1-methyl-4-phenylpyridinium (MPP^+^). Of note, MPP^+^ is taken up by dopaminergic neurons and accumulates in their mitochondria, where it inhibits complex I of the electron transport chain and ultimately causes neuronal cell death. In SH-SY5Y cells, GUO given after as long as 24 h after MPP^+^ was able to reduce MPP^+^-induced caspase-3 activity (Pettifer et al., 2007).

Despite *in vitro* evaluations, data from *in vivo* GUO treatment in PD models are scarce. Chronic treatment with GUO (8 mg/kg for 8 weeks) can significantly reduce bradykinesia in proteasome inhibitor (PSI)-treated rats (Su et al., 2009), which is an animal model for slow-onset PD (McNaught et al., 2004). In addition, chronic GUO treatment reduced apoptotic cell death, induced proliferation of neural progenitor/stem cells, and increased the number of tyrosine hydroxylase-positive cells in the substantia nigra in a PSI model of PD (Su et al., 2009). However, the acute effect and efficacy of GUO in reversing motor impairments in rodent models of movement disorders, including catalepsy, tremor, and hemiparkinsonism, have not previously been addressed.

Reserpine administration to rodents is a primary model for assessing potential PD treatments. L-DOPA efficacy was first reported in this model by observing that it improved the reserpine-induced akinetic state (Carlsson et al., 1957). Even though reserpine does not induce dopaminergic neurodegeneration, the model produces key motor disturbances consistent with those of PD; for example, mice show decreased spontaneous locomotor activity, which correlates to hypokinesia in PD. However, although reserpine-induced catalepsy and TJM were fully reversed, GUO treatment had no effect on spontaneous locomotor activity at the doses tested in this study.

Regarding the hemiparkinsonian rats, unilateral 6-OHDA lesions did not reduce, and subsequent GUO treatment did not increase, spontaneous locomotor activity. Conversely, GUO administration before the subthreshold dose of L-DOPA induced, in a dose-dependent manner, contralateral turning behavior that indicates a pro-dopaminergic action (i.e., GUO enhanced the effects of L-DOPA). Moreover, GUO exerted an antidyskinetic effect in rats chronically treated with L-DOPA. Besides L-DOPA beneficial effects, this long-term therapy leads to development of adverse motor responses, named LID. LIDs, in particular, are a great burden that affects 40–50% of PD patients who undergo L-DOPA treatment for 4–6 years (Ahlskog and Muenter, 2001). Taken together, results from reserpinized mice and hemiparkisonian rats demonstrated that GUO did not alter spontaneous locomotor activity. Interestingly, GUO presented antidyskinetic effect (reducing TJMs in mice and AIMs in the LID model in rats), although it was not observed in the higher dose tested (10 mg/kg). Thus, this surprising U-shaped dose-response of GUO efficacy alleviating TJMs and LID will deserve further investigation in the future. Yet, GUO effect on reserpine-induced catalepsy in mice and potentiation of L-DOPA-induced contralateral rotations in 6-OHDA-lesioned rats was observed in all doses tested. Overall, our data provide evidence that GUO treatment may not only improve the motor symptoms of PD but may also have a potential efficacy on L-DOPA side effects. Thus, the potential clinical importance of this finding is significant.

Importantly, we demonstrated the antiparkinsonian efficacy of GUO in a series of PD rodent models, consistent with other A_2A_R antagonists (Vallano et al., 2011). Indeed, istradefylline (Jenner, 2005) has recently been licensed for use in Japan as an adjuvant to L-DOPA treatment for reducing off-times produced by dopaminergic drugs (Mizuno et al., 2010; Kondo and Mizuno, 2015; Müller, 2015). The molecular target of GUO has not yet been fully characterized and no GUO receptor has been identified, though some studies have suggested its existence (Traversa et al., 2002; Volpini et al., 2011). Alternatively, it has been proposed that GUO might function by altering adenosine receptor functioning (Dal-Cim et al., 2011, 2013; Ciruela, 2013; Lanznaster et al., 2016). Hence, the antiparkinsonian efficacy of GUO might in fact be related to A_2A_R function, thus its relationship with A_2A_R blockade should be further investigated as previously done while assessing similar non-dopaminergic approaches to PD therapy (Kase et al., 2003; Coccurello et al., 2004). Therefore, more experimental work is needed to elucidate the mechanism by which GUO exerts its antiparkinsonian action.

In summary, we have shown the remarkable potential of GUO to ameliorate parkinsonian symptoms in experimental animal models of movement disorders. Subject to further development, GUO is likely to become an excellent candidate for a clinical proof-of-concept study for purinergic-based treatment in PD.

## Author Contributions

CM performed *in vivo* experiments and analyzed data; ML-C performed *in vivo* experiments and analyzed data; FN performed experiments; VF-D designed experiments and wrote the paper; CT conceived and supervised the project, designed experiments, analyzed data and wrote the paper; FC conceived and supervised the project, designed experiments, analyzed data and wrote the paper.

## Conflict of Interest Statement

The authors declare that the research was conducted in the absence of any commercial or financial relationships that could be construed as a potential conflict of interest.
